# Aging and urban innovation: a human capital perspective

**DOI:** 10.3389/fpubh.2024.1520834

**Published:** 2025-01-21

**Authors:** Jinghang Cui, Rong Zhou, K. Jason Crandall, Mingxuan Cui, Ruirui Bai, Yi Jia

**Affiliations:** ^1^School of Sport and Physical Education, North University of China, Taiyuan, China; ^2^Center for Applied Science in Health and Aging, Western Kentucky University, Bowling Green, KY, United States; ^3^College of Philosophy, Law & Political Science, Shanghai Normal University, Shanghai, China; ^4^Department of Cognitive Studies, Vanderbilt University, Nashville, TN, United States

**Keywords:** population aging, urban innovation, the generalized method of moments (GMM), human capital, population aging and urban innovation

## Abstract

**Background:**

With population aging, this demographic dividend diminishes, which may have implications for innovation in a region. Understanding the relationship between population aging and innovation is crucial for addressing economic challenges associated with an aging population.

**Methods:**

This study utilized panel data on population aging and innovation from 252 cities between 2005 and 2014. Various estimation methods, including the fixed effects model, the generalized method of moments (GMM), and the mediation model, were used to analyze the data. These methods allowed for a comprehensive examination of the impact of population aging on innovation and the role of human capital in mediating this relationship.

**Results:**

The findings of the study indicate that both the 60-year-old and 65-year-old population significantly hinder innovation. The GMM suggests that innovation is “path dependent,” meaning that past levels of innovation do not alleviate the negative effects of population aging on future innovation. Additionally, the mediation model analysis demonstrates that human capital plays a crucial role in mediating the relationship between population aging and innovation, highlighting the importance of investing in human capital development.

**Conclusion:**

The findings of this research highlight the obstacles that population aging presents to fostering innovation. Overcoming these obstacles necessitates strategic investments in human capital and policies that support innovation. It is imperative for policymakers to implement recommendations that address population aging and encourage innovation in order to navigate the challenges posed by an aging population and promote a vibrant and dynamic economy.

## 1 Introduction

The demographic dividend has played a crucial role in economic growth in China over the past four decades (1). However, with the emergence of population aging, the demographic dividend is no longer present. In seeking new sources of economic growth, China has incorporated innovation as a key factor into its national strategy. Since the National Science and Technology Conference in 2006, when China first proposed the strategic goal of “implementing an independent innovation strategy and building an innovative nation” (2) and after more than a decade of exploration, in 2016 the State Council clearly stated the goals of “leveraging urban innovation resource-intensive advantages” and “building innovative provinces and cities, enhancing the driving force of innovation development,” pushing innovation to new heights (3). In recent years, the national strategic initiative of “mass entrepreneurship and innovation” aims to expand employment, increase residents’ incomes, promote social mobility and equity, and seek economic transformation and upgrading through innovation (4). The key to benefiting from the demographic dividend lies in the people, while the key to gaining an advantage in innovation lies in human capital. The query of whether the aging of the population will hinder innovation, and whether the aging of the population will affect the development of innovation through human resources, are concerns that China’s present innovation approach must confront head-on, as well as inquiries that the scholars still need to delve into.

Research on the relationship between population aging and innovation has long been a focus, with two main viewpoints. On one hand, it is believed as society ages, there is an increase in social responsibilities, a decline in workforce efficiency, and a depletion of intellectual capabilities, leading to a hindrance in the progress of innovation. Guo et al. (5), based on China’s census and United Nations population projections, pointed out the adverse effects of population aging on labor supply, capital accumulation, and core industries. Yao et al. (6), using the dynamic panel data model and the generalized method of moments, studied the relationship between population aging, human capital accumulation, and technological innovation, finding a negative effect of population aging on technological innovation output, mediated by human capital accumulation. On the other hand, it is believed that the net effect of population aging on technological innovation is positive. Wang et al. (7) found that population aging promotes the upgrading of industrial structure by increasing consumer demand, accelerating human capital accumulation, and forcing enterprises to use capital and technology to cope with rising labor costs. Wang et al. (8) also found, through Dynamic Factor Models (DFM), that aging population has a dual effect on technological innovation, with a beneficial boost to labor productivity but also a detrimental strain on human capital. Liu et al. (9) supported this viewpoint with a spatial econometric analysis using provincial panel data, and pointed out regional differences in the effect of population aging on the transformation toward higher levels of industrial structure. It is evident that the relationship between population aging and innovation is complex and cannot be generalized.

The reason for the different conclusions drawn in the above research may lie in a few factors: firstly, the inappropriate selection of indicators to measure innovation. Innovation activities are sustainable, and using technological progress as an indicator of innovation may not be entirely scientific. Secondly, the issue of data sources. The research mentioned above focused on provinces, where population age structure data is relatively easy to obtain. However, China is vast, with significant differences among provinces. As a result, the research on population aging and innovation results may not be precise enough. In comparison with existing literature, this study aims to make breakthroughs in the following three aspects: first, in terms of research questions, it is easy to obtain data on technological progress, so existing studies mostly focus on the relationship between aging population and technological progress. However, due to the difficulty in obtaining innovative data, there are few studies on the relationship between aging population and innovation. By obtaining data related to population age structure and innovation, this study can directly examine the relationship between the two, which is more meaningful for innovation research and aging research. Secondly, in terms of research scope, the existing studies have mostly focused on national overall, a particular region, or city innovation issues, while this research unit covers data from 252 prefecture-level cities nationwide. Starting from the city-level perspective, it fills the gap in existing research units that are so small that the research questions lose universal significance, or the research scope is too macro and overlooks the lack of heterogeneity within regions. Third, in terms of research content, there are few studies on the relationship between aging population and innovation in existing literature, and no consistent conclusions have been reached. Most literature focuses on the effect of aging on human capital, industrial structure, and technological innovation, but there is a lack of attention to how aging population affects urban innovation through mediators such as human capital.

The structure of the remaining part of this paper is as follows: The second section formulates the research hypothesis of this paper based on theoretical research and literature review; the third section explains the econometric model, variable indicators, and data sources employed in this study; the fourth section uses the model to test the research hypothesis; and the fifth section conducts a mechanism analysis on the outcomes to gain a deeper understanding of the intrinsic connection between population aging and urban innovation, with the aim of refining and delving deeper into the research.

## 2 Theoretical analysis and research hypothesis

In his work “Theory of Economic Development” (10), Schumpeter described development as historical process of structural changes, substantially driven by innovation and that “carrying out innovations is the only function which is fundamental in history” [(11), p. 102]. Subsequently, the “Theory of Innovation” gave rise to both the Linear Model of Innovation (LMI) and the National System of Innovation (NIS). The LMI posits that innovation activities progress linearly, with research and development (R&D) serving as the wellspring of innovation. The relationship between innovation activities and R&D investment is directly proportional, thus innovation capacity can be to some extent simply understood as the level of R&D input (12). However, the LMI fails to rationally explain the disparities in innovative utility produced by the same level of R&D input, leading to the emergence of the NIS. According to Freeman (13), a national innovation system is defined as “the network of institutions in the public and private sectors whose activities and interactions give rise, import, modify, and diffuse new technologies.” Subsequently, the understanding and acceptance of innovation as a “non-linear, interactive system process” has gradually increased. Various elements endowed with innovative resources have been incorporated into the network of the innovation process. The agents of innovation activities have expanded from the micro-enterprise level to the meso-level of cities, regions, and even the macro-level of nations. Their roles are also manifested in broader contexts like the industrial value chain, regional innovation, and global competition, among others.

Existing studies primarily focus on the following aspects: Firstly, examining the relationship between age and innovation from a micro perspective, seeking to identify the peak period of individual innovation; Secondly, exploring how population aging from a macro social perspective impacts the structural elements of innovation, thereby influencing societal innovation; Lastly, investigating the mediation mechanism through which population aging affects innovation activities, i.e., what is the mediator variable that affects innovation due to the aging population?

From the perspective of the innovation lifecycle of individual micro-entities, aging is accompanied by a decline in economic activity abilities such as reasoning, and episodic memory during the labor process (14). Werding (15) confirms an inversely U-shaped relationship between the proportion of workers in different age groups and productivity which mainly works through the TFP (total factor productivity) channel and is significantly stronger than what can be observed at a micro level.

From a holistic perspective of society, population aging will affect the input of elements for social innovation. The effects of population aging on innovation can be categorized as direct effects and indirect effects. On one hand, based on the linear model theory of innovation, from the perspective of input–output, aging affects important resources such as labor supply and savings rate as endowments for R&D, directly affecting innovation activities. On the other hand, the NIS theory, which systematizes the dynamic network of innovation activities, suggests that when the aging population completely withdraws from the production sector and becomes pure consumers, the government’s financial burden for eldercare will increase. Aging threatens innovation input through indirect means such as tax rates, government budget expenditures, and retirement age adjustments. There are two main ways to alleviate financial pressure, with the first being to increase corporate taxes to ease the burden on social old-age support. However, increasing tax burdens will inevitably erode the funds for enterprise technological innovation and limit the enhancement of innovative capabilities. Secondly, in terms of the components of the system, increasing expenditures on social old-age security and healthcare will inevitably reduce investment in other public projects. If investment in research and development and education is compressed, it will have negative effects on human capital accumulation and labor force endowment (7). Therefore, it can be seen that under the current conditions of “getting old before getting rich,” the effects of population aging on innovation are more harmful than beneficial. Therefore, this paper proposes hypothesis 1:

*Hypothesis 1*: All other factors being equal, an aging population is not conducive to innovation.

Although aging weakens the innovation capacity of workers, mature labor force, with knowledge and skills acquired through years of experience, can more effectively promote production and services (16). Jones (17) has pointed out that “great minds produce their greatest insights at substantially older ages today than they did a century ago.” Older workers applying their knowledge and experience to the development of new technologies and products can help improve productivity (18). Lundborg et al. (45) confirmed that learning-by-doing can be a powerful driver of productivity growth in high-skilled occupations. Siliverstovs et al. (19) further studied that the aging of the labor force has a selective mechanism on the utility of innovation activities, with aging negatively affecting employment in the primary and secondary industries but having a positive effect on employment in the tertiary industry, especially in finance and personal services. Higher education levels and abundant work experience can bring more benefits to society and stronger competitiveness to regions (14). Furthermore, the increase in average life expectancy has raised the return on investment in education, while delaying entry into the labor market allows for compensation through improved labor productivity and competitiveness (20). Although the “bottom aging” resulting from declining fertility rates has exacerbated the severity of aging, the decrease in the child dependency ratio concentrates family and societal educational resources on a smaller population, leading to an overall increase in the average level of education and a significant improvement in the quality of the future labor force. Strulik et al. (21), using a micro-founded theory, predict that the previously positive relationship between population growth and productivity growth has turned negative in the 20th century. The Beckerian theory of the child quantity-quality trade-off also offers a similar explanation: when there is a strong substitution relationship between population quantity and quality, the increase in human capital can offset the adverse effects of the decline in population size on innovation (22). The current population structure in China is spindle-shaped, although there is a trend of upward movement, it has not formed an inverted pyramid shape. Currently, there is a higher concentration of people in the working age group. According to “The Human Capital Report 2016,” from 1982 to 2014, the average age of the labor force in China increased from 32.04 years to 35.83 years. In terms of the lifecycle of innovation, the period between 35 and 40 years is considered the golden age for individual innovative activities. Currently, China’s average labor force age has entered the phase of high-frequency innovation, with the accumulation of human capital adding impetus to urban innovation (23, 24). The slight shift in the age structure of the labor force due to aging has enhanced the vitality of social human capital and urban innovation. Based on this, the following hypothesis is proposed:

*Hypothesis 2*: With the increase in the proportion of older population, the human capital stocks of a region will continue to rise.

In terms of the innovation mediation effects, the increase in general human capital and specific human capital both have a stimulating effect on innovation. The aging of the population age structure implies a decrease in the labor force participation in economic activities, and the aging of the labor force population structure (25). Yao et al. (6), using 10 years of provincial panel data in China, employed dynamic panel models and the GMM method, and concluded that population aging has a negative effect on technological innovation. It was further established that societies with higher human capital stocks experience greater efficiency and outcomes in innovation. Human capital stocks serve as a mediator in how aging affects technological innovation, whether aging weakens human capital stocks is crucial in determining the effects of aging on innovation. Nelson et al. (26) pointed out in their research that a nation’s ability to introduce and utilize new technologies depends on the stock of human capital within the country. The more abundant the human capital stocks, the more evident the progress in technological advancements. Bloom and Williamson (27), after incorporating demographic variables into empirical models of economic growth, found that the increase in the proportion of the labor force in East Asian economies from 1965 to 1990 promoted the improvement of per capita productivity, while the subsequent aging of the population brought significant pressure to bear on East Asian economies. In a study on the age structure of workers in various industries in France, Roger et al. (28) discovered a negative correlation between the intellectual and physical supply of workers, labor productivity, and the age structure of workers. Workers aged 40 to 49 make the highest contribution to Total Factor Productivity (TFP), and changes in the age structure of the labor force can affect the TFP growth trends of different countries. According to the overlapping generations model of endogenous economic growth, population aging results in labor becoming relatively scarce compared to capital, and the diminishing marginal returns from capital replacing labor mean that the negative effects on the economy from a decrease in the working-age population cannot be offset by increasing capital investment (29).

The tightening labor supply resulting from population aging signifies the irrefutable reality that the era of unlimited labor supply is gone for good. However, in the current demographic structure, education system, and talent development models, the supply curve of low-skilled labor is declining. The diminishing quantity-based demographic dividend has not led to an increase in the proportion of knowledge-based labor. The benefits of innovation quality-based demographic dividend lag behind the pace of population aging development. The mechanism for promoting human capital upgrading efficiency through aging has not yet been established. Therefore, this paper seeks to put forward Hypothesis 3, aiming to provide a more in-depth validation of the mechanism by which population aging affects urban innovation.

*Hypothesis 3*: Population aging strengthens its inhibitory effect on innovation through human capital.

## 3 Research design

### 3.1 Quantitative model

The central objective of this study is to validate the relationship between the level of population aging and innovation. Based on the aforementioned theoretical hypotheses, the following quantitative model is constructed:


(1) $$\mathrm{in}n_{\mathrm{it}}=\alpha +\beta age_{\mathrm{it}}+\gamma Z+\mu_{i}+v_{i}+\xi_{\mathrm{it}}$$

where *i* represents the city, t represents the time (*year*); $\mathrm{in}n_{\mathrm{it}}$ is the innovation output of city *i* in year *t*; $\mathrm{ag}e_{\mathrm{it}}$ measures the degree of population aging in city *i* in year *t*; Z represents the matrix of other control variables that affect urban innovation; $\mu_{i}$ represents unobserved effects specific to the region, such as differences in regional resource endowments; $v_{i}$ represents unobserved effects specific to time, representing factors such as population policies in different periods; $\xi_{\mathrm{it}}$ represents the random error term.

### 3.2 Variable selection

Response variable: urban innovation index (*Inn*). Regarding the measurement of innovation activities, existing literature commonly employs three indicators: total factor productivity (TFP), R&D expenditure, and the number of patents (Table 1). However, all three metrics have certain drawbacks. In imperfectly competitive markets, measuring innovation using the portion of economic growth that cannot be explained by factor accumulation leads to significant errors. Furthermore, the R&D indicator suffers from substantial inaccuracies due to data availability issues, imperfect accounting systems, and the presence of inflated expenses. In contrast, patent data tend to exhibit higher quality, as they are publicly available, objective, and timely updated, and the nationwide patent confirmation system regulations are largely consistent (30). Moreover, the accessibility of data sources provides a reliable basis for regional disparities in innovation. Nevertheless, relying solely on the quantity of patents to characterize regional innovation has its limitations (31). The growth of innovation output is not solely attributed to the most recent innovation activities but is rather a combined result of past innovation stock and current innovation development (32). Therefore, solely relying on patent data from a given year is insufficient to reflect the actual level of regional innovation. Therefore, this paper, based on the selection of patent data as an alternative indicator of urban innovation capacity, differs from previous studies that directly quantify the number of patents. The patent data utilized in this study are sourced from the “China Urban and Industrial Innovation Index Report.” The distinguishing feature of this report lies in its meticulous consideration of the value disparities among patents based on age. Utilizing the average patent value as a foundation, the urban innovation index is constructed, using the patent-granting invention patents from the National Intellectual Property Administration. The patent valuation is estimated through a patent updating model, and the values of individual patents are aggregated at the city level, thereby obtaining the innovation index for that city (The calculation of the Innovation Index is based on Appendix B1 of the “Report on the Innovativeness of Chinese Cities and Industries”).

**Table 1:** Analysis of indicators related to the “Innovation Index” by Chinese scholars.

Major dimension	Leading scholars	Summary
TFP	Tang (41)	The role of technological innovation is accounted for using the variable growth component of factor income shares.
R&D	Shen & Lu (42)	Regional innovation investment is mainly divided into two indicators, R&D personnel and R&D stock, where R&D personnel is measured by the full-time equivalent of R&D personnel, while R&D capital stock is accounted for by the perpetual inventory method.
	Yu & Zhang (43)	Innovation investment is divided into independent innovation investment and imitation innovation investment. Independent innovation investment uses R&D investment/GDP proxy (%), while imitation innovation investment uses (technology introduction + funding)/GDP proxy (%).
Number of patents	Yao et al. (24)	Measuring the regional innovation index using the number of domestic patent applications granted
	Zhuo et al. (44)	Patents are used to characterize regional innovativeness, with weights of 0.5, 0.3 and 0.2 assigned to invention patents, utility patents and appearance patents, respectively, to measure the innovation index as a weighted average.
	Wang et al. (8)	The number of patent applications is used to construct a technological innovation stock indicator through the perpetual inventory method.

Core explanatory variables: Population aging (*age*). Common indicators used to measure population aging include the aging coefficient, the old-to-young ratio, the old-age dependency ratio, average age, and median age (33). By the United Nations standards for aging societies, this study employs two indicators—the proportion of individuals aged 60 and above in the total population (*age60*) and the proportion of individuals aged 65 and above in the total population (*age65*)—to calculate the population aging coefficient. While provincial-level population aging data is relatively easy to access, obtaining municipal-level data on population aging poses challenges. Previous research on urban population aging has predominantly drawn upon age-grouped data from population censuses and sampled survey data. However, this data is only available for specific survey years, limiting its use to cross-sectional studies and hindering the creation of panel data. The population aging data for this study is sourced from the Chinese Center for Disease Control and Prevention. Initiated in 2004, this database is based on the population data released by the National Bureau of Statistics of China and has been cross-checked and adjusted by each province. To facilitate the annual calculation of disease incidence rates across age groups, the database provides detailed statistics on population breakdown by age and region. This study obtained the proportion of older population in various prefecture-level cities from this database, which currently only discloses age group data for the residential population at the city level from 2005 to 2014.

In addition to the core explanatory variables, considering the factors that influence innovation, as well as relevant literature (34, 35), we have selected the following variables as control variables in terms of macroeconomics, social structure, and population quality.

Initially, from a macroeconomic perspective, the examination of the effects of national finance and industrial structure on innovation was conducted, utilizing the ratio of government expenditure to fiscal revenue (*selffin*), the proportion of the secondary industry (*second*), and the proportion of the tertiary industry (*third*) as control variables. Technological innovation is the key to a country’s advancement in economic development to its pinnacle, while the government’s productive public expenditure directly affects the economic production domain. Major developed countries globally are continuously enhancing public research and development investment, fostering societal innovation activities through fiscal support. The transformation of the industrial structure from labor-intensive to technology and knowledge-intensive is recognized as a significant manifestation of innovation. From a supply-driven perspective, an excessive proportion of the secondary industry may lead to a deficiency in innovation upgrading capabilities. The innovative sectors mostly belong to the tertiary industry, with innovation activities exhibiting a noticeable positive effect on the tertiary industry, facilitating the advancement of the industrial structure toward sophistication. Conversely, the development of the tertiary industry will stimulate the emergence of innovative activities tailored to industrial demands.

Next, from a perspective of social structure, factors such as population density, the number of library books, and wages were examined in relation to the effects of urban innovation. Population density (*lnpopren*) is an important indicator of regional population distribution, laying the foundation for the overall social population structure of a region. According to Boserup (36), an increase in population density leads to progress in technological innovation, with an increase of one standard deviation in population density resulting in 1.5 more patents per ten thousand employed individuals. On the other hand, the number of library books per 10,000 people (*lnlib*) is a reflection of the emphasis on knowledge and social investment in education in a region. Regions with a great number of library books typically experience a fast diffusion of innovations and also have higher stocks of knowledge-based human capital (37).

Finally, the quality of the population is a soft condition that affects innovation. According to the Petty-Clark Theorem, as the economy develops, labor shifts from agriculture to manufacturing, but as per capita income further increases, labor will then shift toward the tertiary industry (38). Therefore, average wages (*wage*) are closely related to the development of regional innovative industries, with workers in technology-intensive industries usually able to earn higher wages. Viewing innovation as a form of production, regions with high human capital stocks will have high innovation efficiency and outcomes, with education and experience being key characteristics of human capital (39). The number of university students enrolled (*lnstu*) reflects the knowledge accumulation gained from formal education, laying the foundation for a versatile talent base for innovation, an important human resource potential for innovation activities. Meanwhile, urban areas having the number of hospital beds (*lnmed*) represent the region’s population health status, an indicator of population health quality. Good health contributes to prolonging the duration during which workers engage in innovative activities. The overall healthiness of society plays a role in bolstering the scale of intellectual human capital, providing a solid foundation for the effective utilization of knowledge-based human capital. Per capita consumption (*lnpsum*) reflects the projection of hotspots in the consumption market across the entire industry level in modern society. To increase efficiency and seize market share, businesses will increase their innovative investments in these consumption hotspots, causing heterogeneous effects on the development of various industries. The higher the consumption level, the stronger the drive for innovation.

In addition to the aforementioned variables, this paper seeks to analyze the pathway through which population aging affects innovation by examining the following mediator variables. The fundamental components of innovation are rooted in human capital, with human capital representing the knowledge and skills possessed by individuals and serving as the “capacity” and “driving force” of innovation (6). Human capital serves as a crucial input in innovative endeavors, with a greater total volume of human capital and higher quality of human capital per capita indicating a stronger foundation of talent for innovation development.

### 3.3 Data sources

The response variable “innovation index” in this study is obtained from the “Report on China’s Urban and Industrial Innovation Capability.” The data on population aging is derived from the “Infectious Diseases Network Report Population Database,” which calculates data on age groups of the permanent population in different prefecture-level cities. Since the data in this database was only available up to 2014, the study period for this paper spanned from 2005 to 2014. The data on human capital per capita and total human capital stocks are sourced from the “China Human Capital Report (2017),” which measures human capital levels using the Jorgenson-Fraumeni lifetime income approach. This approach provides a more comprehensive assessment of regional human capital status and the long-term effects of investments in education and health. Other control variables are obtained from the “China Regional Economic Statistical Yearbook” and the “China Urban Economic Statistical Yearbook.” Due to missing statistical data in some regions, a sample matching technique was used to determine a final research sample of 252 prefecture-level cities. The study period spanned from 2005 to 2014.

Table 2 presents the descriptive statistics of variables in this article. Regarding the spatial distribution between population aging (*age60*) and urban innovation (*inn*) in China from 2005 to 2014, it can be seen that population aging exhibits significant unevenness. The city with the highest population aging rate reaches 24.801%, with an average of 12.442% over the decade, exceeding the social standard of population aging with a 10% proportion of older population. Urban innovation also shows significant spatial distribution differences, with the city with the highest innovation index at 6.504 and the city with the lowest at 0. The national average innovation index for cities is 0.899.

**Table 2:** Descriptive statistics.

Variables	Description of variables	Sample size	Average value	Standard deviation	Minimum value	Maximum values
Explanatory variable						
*lninn*	Urban innovation index (logarithmic)	2,510	0.899	1.031	0	6.504
Core explanatory variables						
*age60*	Percentage of older adults over 60 (%)	2,510	12.442	2.414	2.864	24.801
*age65*	Percentage of older adults over 65 (%)	2,510	8.317	1.669	1.739	17.035
Control variable						
*lnlib*	The number of library books per 10,000 people (logarithm)	2,510	3.943	0.966	0	7.691
*lnmed*	The number of hospital beds in the urban area (logarithm)	2,510	3.833	0.569	1.523	5.541
*lnpopren*	Population density (logarithm)	2,510	13.808	0.763	11.874	16.700
*lnpsum*	Per capita consumption (logarithm)	2,510	9.176	0.796	5.748	11.650
*second*	The percentage contribution of the secondary industry	2,510	51.174	12.753	8.050	90.970
*selffin*	The ratio of government expenditure to government revenue	2,510	2.179	2.263	0.048	43.844
*lnstu*	The number of university students enrolled (logarithm)	2,510	5.684	1.008	0.010	8.367
*third*	The percentage contribution of the tertiary industry	2,510	40.374	10.763	8.580	78.660
*lnwage*	Per capita wage (logarithm)	2,510	9.998	0.482	8.827	11.743
Mechanism explanatory variables						
*lnrphc*	Total human capital stock (logarithm)	2,510	11.998	0.349	11.049	13.239
*lnrplhc*	Human capital stock per capita (logarithm)	2,510	11.445	0.350	10.669	12.776

## 4 Results analysis

### 4.1 Regression benchmarking

The Equation (1) was first utilized to examine the effects of population aging on urban innovation without addressing endogenous variables. The method of least squares was first utilized to examine the effects of population aging on urban innovation without addressing endogenous variables. All four models controlled for time fixed effects and city fixed effects, and were found to be reasonable through F-tests. The adjusted R-squared values for all four models were above 0.65, indicating that the explanatory power of the models was over 65%.

In Table 3, column (1) solely addresses the relationship between population aging at 60 years and above and urban innovation. The results indicate that population aging has a significant negative effect on urban innovation at the 1% level, suggesting a pronounced detrimental effect of population aging on urban innovation. Specifically, for every 1% increase in population aging, the urban innovation index is projected to decrease by 2.2%, thereby verifying hypothesis 1. In columns (2)–(4), upon incrementally introducing different control variables, the significance levels improve consistently through 1% significance tests. Notably, the coefficient of population aging remains relatively stable, indicating the consistent hindering effect of population aging on urban innovation.

**Table 3:** Regression benchmarking.

Explanatory variable	Explained variable: *inn*			
	(1)	(2)	(3)	(4)
*age60*	−0.022^***^	−0.017^***^	−0.016^***^	−0.017^***^
	(0.007)	(0.006)	(0.006)	(0.006)
*selffin*		0.022^**^	0.021^***^	0.018^**^
		(0.008)	(0.008)	(0.007)
*second*		−0.011^***^	−0.010^***^	−0.008^**^
		(0.004)	(0.004)	(0.003)
*third*		0.005^*^	0.006^*^	0.007^***^
		(0.003)	(0.003)	(0.003)
*lib*			0.051^**^	0.058^***^
			(0.021)	(0.021)
*popren*			0.529^***^	0.395^***^
			(0.135)	(0.132)
*wage*				−0.085^*^
				(0.045)
*stu*				−0.115^***^
				(0.023)
*med*				−0.058
				(0.047)
*psum*				0.001
				(0.032)
_cons	0.675^***^	0.937^***^	−6.627^***^	−3.311
	(0.076)	(0.289)	(1.931)	(2.121)
*N*	2,510	2,510	2,510	2,510
Time fixed	Y	Y	Y	Y
City fixed	Y	Y	Y	Y
Adj_R^2^	0.652	0.682	0.694	0.708
F	96.914	73.160	64.515	56.332

Robust standard errors in parentheses, “^*^,” “^**^,” and “^***^” represent significance levels at 10, 5, 1%, respectively.

From other control variables, column (2) mainly examines the effects of urban innovation from a macroeconomic perspective by considering the country’s fiscal situation and industrial structure; column (3) explores the effect of social structures such as population density and the number of library books per 10,000 people on urban innovation; and column (4) includes factors such as the number of university students enrolled, the number of hospital beds, wages, and other indicators related to labor quality to analyze the effects of population quality on urban innovation. Based on the estimated results of the model, there are three aspects of analysis.

From a macroeconomic viewpoint, the governmental fiscal balance exhibits a positive relationship with urban innovation, signifying that higher fiscal expenditure is conducive to the advancement of urban innovation. Nonetheless, the coefficient of the fiscal balance ratio ranges merely from 0.018 to 0.022%, indicating a relatively modest promoting effect of fiscal balance on urban innovation. There exists a notable correlation between the proportions of the secondary and tertiary industries and urban innovation. The concentration of labor-intensive manufacturing activities constrains the realization of urban innovation potential, as evidenced by a 1% increase in the secondary industry proportion correlating with a decline of 0.8 to 1.1% in the innovation index. However, upon the incorporation of a labor quality indicator in Column (4), the coefficient associated with this relationship experiences a decrease in significance. This could potentially be attributed to the fact that an enhancement in labor quality heightens selectivity in employment, resulting in a reduction in the number of laborers engaged in the secondary industry. The obstruction posed by the secondary industry on innovation is mitigated through the enhancement of labor quality. Conversely, for every 1% increase in the proportion of the tertiary industry, the innovation index is projected to increase by 0.5%. While the driving force of the tertiary industry may be relatively weaker compared to the constraints of the secondary industry, the inclusion of the labor quality variable leads to a substantial increment in the coefficient and significance associated with the tertiary industry. The augmentation of labor quality bolsters the positive effect of the tertiary industry on urban innovation.

From a perspective of social structure, the number of library books per 10,000 individuals and population density demonstrate significant correlations with urban innovation, both with a positive effect. In Column (3), the correlation between the number of library books per 10,000 individuals and urban innovation is confirmed through a 5% significance test, with a coefficient of 0.051. Upon the inclusion of the labor quality variable in Column (4), this relationship passes a 1% significance test, resulting in a slight increase in the coefficient to 0.058. Furthermore, the significance of the relationship between population density and urban innovation is also confirmed through a 1% significance test. For every 1% increase in population density, the effect on innovation escalates by 0.395%. Upon the incorporation of the quality of the population variable, the estimated coefficient rises to 0.529%. Hence, it is evident that mere urban population growth does not exert a significant effect on urban innovation. The primary driving force behind innovation stems from the aggregation of highly skilled individuals within urban areas.

From the perspective of the quality of population index, in Column (4), both average wages and the number of university students enrolled show significant correlations with urban innovation. The correlation between average wages and urban innovation is negative; a 1% increase in average wages leads to a decrease of 0.085% in urban innovation, contrary to the expected hypothesis. One possible reason for this is that, although according to the Petty-Clark theorem, an increase in income would lead workers to shift toward higher-level industries, the current situation in China still sees over half of the labor force employed in primary and secondary industries. According to statistics from the National Bureau of Statistics, the proportion of employment in the primary industry was 27.70% in 2016, the secondary industry was 28.80%, and the tertiary industry accounted for 43.50%. Despite the increase in labor wages, a significant portion of workers remain concentrated in non-innovation-intensive industries, hampering the development of urban innovation. On the other hand, the number of university students enrolled shows a significant negative correlation with urban innovation at a 1% level of significance. When per capita consumption increases by 1%, the urban innovation index decreases by 11.5%, which deviates from the expected hypothesis. This may be because university students are in the stage of knowledge accumulation and are not yet active labor and innovation practitioners, but rather serve as potential labor and innovation backup forces. A higher number of university students in a region implies a larger population of delayed job seekers who have not yet entered the labor force, thus leading to a relatively smaller labor force in the region. However, these individuals represent high-quality human resources for the future job market and are also the backbone of future technological innovation, playing a crucial role in future innovative activities. The number of hospital beds per capita in urban areas and per capita consumption shows no significant correlation with urban innovation. However, the coefficients of other variables remain unchanged, and there is an increase in the r-squared value of the model, indicating an enhancement in the explanatory power of the model. As control variables were gradually introduced from Step (1) to Step (4), the relationship and significance of population aging with the urban innovation index did not significantly change. This suggests that the negative effect of population aging on urban innovation is robust and that population aging has an unfavorable effect on urban innovation.

### 4.2 Robustness testing

In this section, we use the proportion of the population aged 65 and above (*age65*) instead of the proportion of the population aged 60 and above (*age60*) for robustness testing. The study found that under the population aging standard of 65 years old, population aging still has a significant negative effect on innovation activities. In all four models in Table 4, the coefficient of population aging passed the significance test at the level of 1% or higher. In column (1), when considering only population aging as an indicator, a 1% increase in population aging led to a 3.3% decrease in the urban innovation index compared to the benchmarking model, with an increase of 1.1% in the value. Furthermore, after gradually adding control variables, the coefficients were all higher than those in the benchmarking model, indicating that as the proportion of the population aged 65 and above increased by one percentage point, the problem of population aging became more serious, resulting in a greater negative effect on innovation. In column (2), after adding three macroeconomic indicators as control variables, the regression results slightly improved compared to the benchmarking model, showing that government fiscal expenditure and the transformation of industrial structure play a positive role in promoting urban innovation. Similarly, in column (3), after adding social structure variables, the estimation results remained consistent with Table 2, with no change in significance level and a slight increase in the coefficient of the interaction term. Finally, in column (4), after adding all control variables, the estimation results remained highly robust, with the basic estimation results of the benchmarking model unchanged. It can be confirmed that the estimation results of the above variables all passed the robustness test.

**Table 4:** Robustness testing.

Explanatory variable	Explained variable: *inn*			
	(1)	(2)	(3)	(4)
*age65*	−0.033^***^	−0.026^***^	−0.025^***^	−0.025^***^
	(0.008)	(0.007)	(0.007)	(0.007)
*selffin*		0.022^***^	0.021^***^	0.018^**^
		(0.008)	(0.008)	(0.007)
*second*		−0.011^***^	−0.010^***^	−0.008^**^
		(0.004)	(0.004)	(0.003)
*third*		0.006^*^	0.006^**^	0.008^***^
		(0.003)	(0.003)	(0.003)
*lib*			0.051^**^	0.058^***^
			(0.021)	(0.021)
*popren*			0.532^***^	0.402^***^
			(0.136)	(0.133)
*wage*				−0.084^*^
				(0.045)
*stu*				−0.114^***^
				(0.023)
*med*				−0.055
				(0.048)
*psum*				0.001
				(0.032)
*_cons*	0.676^***^	0.910^***^	−6.702^***^	−3.449
	(0.059)	(0.285)	(1.951)	(2.142)
*N*	2,510	2,510	2,510	2,510
Time fixed	Y	Y	Y	Y
City fixed	Y	Y	Y	Y
R^2^	0.653	0.683	0.694	0.708
F	96.984	73.054	64.560	56.305

The values in parentheses represent the robust standard errors. “^*^,” “^**^,” and “^***^” denote significance levels of 10, 5, and 1%, respectively.

### 4.3 GMM estimation

Both regression benchmarking and robustness testing are conducted without considering the issue of endogeneity. Endogeneity refers to while partial effect of an independent variable on a dependent variable is unobservable and it will cause outcomes biased. However, there exists a reciprocal relationship between innovation and population aging. Therefore, the aforementioned study merely establishes the correlation between population aging and innovation, without being able to establish a causal link between the two. Furthermore, innovation activities exhibit continuity, with current innovation activities being influenced by past innovation outcomes. In such situation, the delayed effects of past innovation are regarded as omitted endogenous factor of variables. Hence, it is essential to introduce lagged variables and transform the benchmarking model into a dynamic model. The model is set up as follows:


(2) $$\mathrm{in}n_{\mathrm{it}}=\alpha +\overset{n}{\underset{n=1}{\sum }}\lambda_{n}\mathrm{in}n_{\mathrm{it}-n}+\beta age_{\mathrm{it}}+\gamma Z+\mu_{i}+\nu_{t}+\xi_{\mathrm{it}}$$

where $\mathrm{in}n_{\mathrm{it}-n}$ represents n lagged periods of innovation. In dynamic panel models, the estimation method typically involves the use of GMM. The advantage of this method lies in its capability to address the endogeneity problem of both lagged variables and core explanatory variables. Therefore, employing GMM enables the estimation of the causal relationship between population aging and innovation.

According to Equation (2), Table 5 shows the estimation results of GMM, with all four models controlling for one lagged period of the innovation index, as well as time and region fixed effects. According to the examination of AR(1) and AR(1) tests, it is observed that there is first-order serial correlation but no second-order serial correlation present. The non-significance of the Hansen test indicates the reliability of the GMM estimation results. Among the four models, the one lagged period of innovation significantly influences current innovation positively. In column (1), without the inclusion of control variables, population aging exhibits a significant negative effect on urban innovation. For every 1% increase in the proportion of the population aged 60 and above, the urban innovation index decreases by 1%. Upon the introduction of control variables in column (2), the significance is confirmed through a 1% level of significance test, with the urban innovation index decreasing by only 0.4%. In columns (3) and (4), where the proportion of the population aged 65 is used as an explanatory variable, population aging has a significant negative effect on urban innovation without control variables, with a coefficient of −0.013. This indicates that a 1% increase in the proportion of the population aged 65 leads to a 1.3% decrease in the urban innovation index, slightly higher than the standard of 60 years of age for population aging. However, upon adding all control variables, the significance between the two variables significantly increases and passes the 1% level of significance. The coefficient slightly decreases to 0.5%. Overall, while controlling for fixed time and urban factors, the inclusion of lagged variables leads to changes in the significance and effect of population aging on urban innovation under different conditions. Nonetheless, the obstructive effect of population aging on urban innovation persists.

**Table 5:** The results of the SYS-GMM estimation.

Explanatory variables	Explained variable: *inn*			
	(1)	(2)	(3)	(4)
*L.inn*	1.078^***^	1.021^***^	1.079^***^	1.022^***^
	(0.009)	(0.020)	(0.009)	(0.020)
*age60*	−0.010^*^	−0.004^***^		
	(0.005)	(0.001)		
*age65*			−0.013^*^	−0.005^***^
			(0.007)	(0.001)
constant term		−0.649^**^		−0.624^**^
		(0.304)		(0.305)
*N*	2,259	2,259	2,259	2,259
Control variable	N	Y	N	Y
Time fixed	Y	Y	Y	Y
City fixed	Y	Y	Y	Y
Wald chi2	126596.25	66585.92	137355.67	66426.92
AR(1) *p*-value	0.001	0.000	0.082	0.007
AR(1) *p*-value	0.874	0.142	0.965	0.139
Hansen *p*-value	0.245	0.190	0.809	0.202

Robust standard errors are reported in parentheses. “^*^,” “^**^,” and “^***^” denote statistical significance at the 10, 5, and 1% levels, respectively.

## 5 Further discussion

This section will focus on discussing the mediation mechanism through which population aging affects urban innovation, specifically how population aging indirectly influences urban innovation through human capital. This study posits that population aging may indirectly influence urban innovation through human capital. To examine the mediation effects of human capital, drawing upon the Baron and Kenny Method of Testing Mediation (40), the following steps are undertaken: ① Test the effects of population aging on urban innovation. If the coefficient of population aging is significant, it indicates a significant effect of population aging on urban innovation and proceeds to the next step of testing; ② Test the effects of population aging on human capital. If the coefficient of population aging is significantly positive, it suggests that an increase in population aging is beneficial for the enhancement of urban human capital; ③ Based on step one, gradually introduce human capital variables. If the effects of the mediator variable are positive and the coefficient of population aging relative to step one decreases or becomes non-significant, it indicates that human capital has partial or even full mediation effects.

According to the above testing method, we have established the following empirical model:

First, test if population aging affects urban innovation.


(3) $$\mathrm{In}\;n_{\mathrm{it}}=\beta_{0}+\beta_{1}\;{\mathrm{age}}_{\mathrm{it}}+\gamma Z+\mu_{i}+\nu_{t}+\xi_{\mathrm{it}}$$

Second, examine whether population aging affects human capital.


(4) $$hc_{\mathrm{it}}=\beta_{0}+\beta_{2}\;\mathrm{ag}e_{\mathrm{it}}+\gamma Z+\mu_{i}+\nu_{t}+\xi_{\mathrm{it}}$$

Third, include both the population aging variable and the human capital variable in the model.


(5) $$\mathrm{In}n_{\mathrm{it}}=\beta_{0}+\beta_{1}\mathrm{ag}e_{\mathrm{it}}+\beta_{3}hc_{\mathrm{it}}+\gamma Z+\mu_{i}+\nu_{t}+\xi_{\mathrm{it}}$$

where *hc* represents human capital, while *Z* represents control variables.

According to Equations (3–5), Table 6 shows the estimation results of the logarithm of total human capital as the mediator variable. The first three columns present the estimation results using the proportion of the population aged 60 and above as core explanatory variables. The results indicate that population aging has a significant negative effect on urban innovation; population aging has a significant positive effect on the logarithm of total human capital. When simultaneously examining human capital and population aging, human capital shows a significant positive effect on innovation, while population aging continues to exhibit a significant negative effect on innovation, with a smaller coefficient (more negative), suggesting that population aging indeed influences human capital, thereby affecting innovation. Human capital serves as the mediation variable between population aging and innovation, confirming hypotheses 2 and 3. When using the proportion of the population aged 65 and above to measure population aging, the coefficient in the first step is −0.025, while in the third step it is −0.028, indicating the robustness of the mediation effects on total human capital. Overall, with the inclusion of human capital as the mediator variable, the negative effect of population aging on urban innovation is expected to increase by 0.3–0.4%.

**Table 6:** Testing for mediation effects of total human capital.

Explanatory variables	Mediation effects of total human capital (age60)			Mediation effects of total human capital (age65)		
	First step	Second step	Third step	First step	Second step	Third step
	*inn*	*rphc*	*inn*	*inn*	*rphc*	*inn*
*age60*	−0.017^***^	0.003^***^	−0.021^***^			
	(0.004)	(0.000)	(0.004)			
*age65*				−0.025^***^	0.003^***^	−0.028^***^
				(0.005)	(0.001)	(0.005)
*Rphc*			1.254^***^			1.204^***^
			(0.167)			(0.166)
constant term	−3.311^***^	11.812^***^	−18.127^***^	−3.449^***^	11.851^***^	−17.722^***^
	(1.019)	(0.127)	(2.217)	(1.016)	(0.128)	(2.211)
*N*	2,510	2,510	2,510	2,510	2,510	2,510
Control variable	Y	Y	Y	Y	Y	Y
Time fixed	Y	Y	Y	Y	Y	Y
City fixed	Y	Y	Y	Y	Y	Y
F-test	285.679	4833.282	280.897	286.459	4770.574	281.020
Adj_R^2^	0.673	0.973	0.681	0.673	0.973	0.681

The values in parentheses represent robust standard errors, “^*^,” “^**^,” and “^***^” denote significance levels of 10, 5, and 1%, respectively.

Table 7 displays the results of the model testing the mediation effects with human capital per capita. Regardless of whether using the proportion of the population aged 60 and above or 65 and above, in comparison to the first step, the coefficients of the effects of population aging on urban innovation both decrease in the third step. This indicates that by using human capital per capita as the mediator variable, human capital similarly plays a mediation role between population aging and innovation. When incorporating the human capital variable, the negative effect of population aging on urban innovation increases by 0.5%.

**Table 7:** Testing for the mediation effects of human capital per capita.

Explanatory variables	Mediation effects of human capital per capita (age60)			Mediation effects of human capital per capita (age65)		
	First step	Second step	Third step	First step	Second step	Third step
	*inn*	*rplhc*	*inn*	*inn*	*rplhc*	*inn*
*age60*	−0.017^***^	0.004^***^	−0.022^***^			
	(0.004)	(0.001)	(0.004)			
*age65*				−0.025^***^	0.004^***^	−0.030^***^
				(0.005)	(0.001)	(0.005)
*lnrplhc*			1.518^***^			1.483^***^
			(0.140)			(0.139)
_cons	−3.311^***^	10.949^***^	−19.932^***^	−3.449^***^	10.991^***^	−19.755^***^
	(1.019)	(0.150)	(1.826)	(1.016)	(0.150)	(1.823)
*N*	2,510	2,510	2,510	2,510	2,510	2,510
Control variable	Y	Y	Y	Y	Y	Y
Time fixed	Y	Y	Y	Y	Y	Y
City fixed	Y	Y	Y	Y	Y	Y
F-test	285.679	3691.671	291.425	286.459	3653.940	291.490
Adj_R^2^	0.673	0.965	0.689	0.673	0.965	0.689

The values in parentheses represent robust standard errors, “^*^,” “^**^,” and “^***^” denote significance levels of 10, 5, and 1%, respectively.

## 6 Conclusion

As the demographic dividend diminishes, innovation becomes a new driving force for China to participate in international competition. However, the issue of population aging follows closely. Therefore, exploring the relationship between population aging and innovation is a worthwhile research endeavor. This study utilizes panel data from 252 city-level observations from 2005 to 2014 to investigate the relationship between population aging and urban innovation. Employing fixed effects models and GMM, the study yields the following findings: (1) Using the proportion of the population aged 60 and above as a measure of population aging, the results indicate a significant hindering effect of population aging on urban innovation. When considering the age standard of 65 years, the obstruction of population aging on innovation is even greater. (2) Taking into account the continuity of innovation activities, the one lagged period of innovation activity was controlled, and the estimation results using GMM reveal that innovation is “path dependent.” However, such “path dependency” does not alleviate the hindering effect of population aging on urban innovation. (3) In the analysis of mechanisms, employing the mediation model reveals that human capital serves as a mediator between population aging and urban innovation. This suggests that population aging significantly affects urban innovation and human capital. Furthermore, when simultaneously considering human capital and population aging as control variables, the negative effect of population aging on urban innovation becomes more pronounced.

Based on the conclusions of the aforementioned study, the policy implications of this paper primarily manifest in the following aspects:

Firstly, we must acknowledge the disappearance of the demographic dividend, the exacerbation of population aging, and the challenging circumstances surrounding innovation activities. The rapid development of China over the past four decades has relied crucially on the demographic dividend. However, with its disappearance and the subsequent issue of population aging, China’s demographic concerns have become more severe. Although measures such as the relaxation of the two-child policy have been implemented by the government to alleviate the problem of population aging, it is difficult to swiftly stimulate a long-term suppressed fertility desire through policy interventions. While innovation serves as a source of growth for the new economy, the obstructive effect of population aging on it has been confirmed. It is imperative to properly recognize the relationship between population aging and innovation, mitigate the effects of population aging, and implement suitable measures to enhance innovation capabilities, particularly among the younger population.

Secondly, individuals serve as the foundation of innovation, and enhancing human capital is the cornerstone of innovation. Research findings indicate that population aging has a positive effect on human capital stocks, and human capital, in turn, has a positive effect on innovation. This suggests that the human capital of the older population is an important component of human capital stocks. Given the fundamental solidification of human capital within the older population, which poses challenges for further augmentation, the primary source of overall human capital stock expansion lies in the human capital of the younger population.

Thirdly, innovation is “path dependent,” and nurturing innovation capabilities is a process of long-term accumulation. Therefore, in the discourse of enhancing national innovation capabilities, it is imperative not to rush and instead formulate long-term strategic planning. By fostering internal growth and attracting innovative talents, innovation activities can become a more common choice for the younger generation. Only through this approach can a country or region reconcile the reality of population aging with the vision of innovation-driven development and embark on the path of innovation-boosted progress.

In light of these findings, several avenues for future research and policy development are recommended. Future research should consider longitudinal studies to track the long-term impact of population aging on innovation, providing deeper insights into the temporal dynamics and causal relationships between these variables. Investigating the effectiveness of various policy interventions aimed at mitigating the negative impacts of population aging on innovation, including the impact of fiscal incentives, educational reforms, and immigration policies on sustaining and enhancing human capital, is essential. Exploring the role of emerging technologies such as artificial intelligence, automation, and biotechnology in counteracting the negative effects of population aging will be crucial for understanding how these technologies can complement human capital and drive innovation. Conducting comparative studies across different countries with varying demographic profiles can identify best practices and policy measures that successfully address the challenges of population aging while fostering innovation. Encouraging interdisciplinary research that combines insights from economics, sociology, public health, and urban planning can develop holistic strategies for managing the dual challenges of population aging and the need for continuous innovation. Additionally, developing and implementing innovation policies that are inclusive of all age groups, recognizing the potential contributions of older adults to innovation, and creating supportive environments for lifelong learning and productivity can help mitigate the negative impacts of aging on innovation. By addressing these future directions, policymakers and researchers can better navigate the complexities of an aging population and its implications for innovation, ensuring sustained economic growth and competitiveness.

## Data Availability

The original contributions presented in the study are included in the article/Supplementary material, further inquiries can be directed to the corresponding authors.
